# Modified Nanofibrous Filters with Durable Antibacterial Properties

**DOI:** 10.3390/molecules26051255

**Published:** 2021-02-26

**Authors:** Ganna Ungur, Jakub Hrůza

**Affiliations:** Institute for Nanomaterials, Advanced Technologies and Innovation, Technical University of Liberec, 46001 Liberec, Czech Republic; jakub.hruza@tul.cz

**Keywords:** nanofibers, filtration, polyurethane, copper oxide, microparticles, nanoparticles, electrospinning, antibacterial properties

## Abstract

The main aims of the research were to produce efficient nanofibrous filters with long-term antibacterial properties and to confirm the functionality of samples under real filtration conditions. A polyurethane solution was modified by micro- or nanoparticles of copper oxide in order to juxtapose the aggregation tendency of particles depending on their size. Modified solutions were electrospun by the Nanospider technique. The roller spinning electrode with a needle surface and static wire electrode were used for the production of functionalized nanofibers. The antibacterial properties of the modified nanofibrous layers were studied under simulated conditions of water and air filtration. Particular attention was paid to the fixation mechanism of modifiers in the structure of filters. It was determined that the rotating electrode with the needle surface is more efficient for the spinning of composite solutions due to the continuous mixing and the avoidance of particle precipitation at the bottom of the bath with modified polyurethane. Moreover, it was possible to state that microparticles of copper oxide are more appropriate antimicrobial additives due to their weaker aggregation tendency but stronger fixation in the fibrous structure than nanoparticles. From the results, it is possible to conclude that nanofibers with well-studied durable antibacterial properties may be recommended as excellent materials for water and air filtration applications.

## 1. Introduction

A new class of composite materials based on organic and inorganic species has attracted considerable attention since these materials have the benefits of the organic component, such as light weight, flexibility and moldability, and the inorganic component, such as high strength, heat stability, chemical resistance and different functional properties. Therefore, fabrication of composite nanofibers (NFs) consisting of inorganic materials encapsulated in a polymer matrix has been under intensive investigation in the past few years [1].

Polymeric NFs have been actively used in the commercial air and water filtration applications over the last 30 years [2,3]. The unique properties of nanofibrous layers, such as high porosity up to 90% and a large specific surface area, make them excellent candidates for filtration purposes [4]. The very high surface area of NFs facilitates the adsorption of contaminants from the air. Moreover, the air filtration capability of nanofibrous layers increases for the same pressure drop compared to conventional fiber mats due to the slip flow effect [2,5]. Electrospun membranes have found application in pressure-operated liquid filtration processes, such as microfiltration, ultrafiltration, nanofiltration and reverse osmosis, due to their inter-connective porous morphology and the uniform distribution of nano-pore size [6]. Composite electrospun fabrics have recently been emerged as an effective membrane for removing of harmful air- and water-born contaminants from the environment [3,5].

Electrospinning (ES) is the most effective way to produce nanoscale fibrous webs with small pores, high porosity and good permeability [7]. Furthermore, free surface or needleless ES provides excellent capacity to initiate multiple jets from a charged liquid surface. Several methods have been reported to launch free surface ES, for example, liquid-filled trenches, slits, wetted spheres, rotating and fixed wires, cylinders, discs, conical wire cores, and gas bubbles rising through the liquid surface [8]. The needleless ES Nanospider technique is the most suitable for producing NFs in the mass production range [4,5]. The introduction of inorganic components into the nanofibrous structure may be accomplished by the electrospinning of polymer solutions containing the appropriate particles (the blending method) [6,7,8,9], by the impregnation method [10] or by in-situ reduction of metal salts or complexes into the polymeric matrix [11,12,13]. The blending method has been proven to be an effective and simple method to functionalize NFs without extra technological steps [11].

Polyurethane has been modified by different types of inorganic clusters, such as Ag, CNTs (carbon nanotubes), Zn-Ag bimetallic particles, tourmaline, silica and ZnO. Ag is the most commonly used additive to confer the antimicrobial properties of both natural and synthetic fibers (including PU nanofibers [12,14,15,16]. However, the amount of research focusing on the modification of PU NFs by copper oxide (CuO) NPs is relatively small. In the study [17] the electrical conductivity of electrospun polyurethane mats with NPs of CuO was successfully confirmed. Another study [18] provided data about the good antibacterial properties of PU/CuO NPs layers. CuO is also a very perspective additive for the efficient modification of nanofibers due to its unique biological, antibacterial properties and its low cost of preparation [19]. Moreover, CuO is easily mixed with polymers maintaining physical and chemical stability. Particles of CuO have the potential for external uses such as antibacterial agents in surface coatings of various substrates to prevent microorganisms from attaching, colonizing, spreading and forming biofilms in medical devices [20]. 

Despite the unique functional properties of NPs, their toxicity and tendency to aggregate still raise many questions about the safety of nanoscale metal oxides and technological rationality to deal with unstable systems. It has been proven that NPs induce different levels of cytotoxicity and DNA damage [21]. Moreover, nanoparticles have a strong tendency to undergo agglomeration followed by insufficient dispersal in the polymer matrix, degrading the functional properties of nanocomposites [22].

There is a key question about the stability of the insertion of antimicrobial substances in order to prove the durability of the antibacterial properties and safety of filters, especially for water filtration. Therefore, the parsing of the control methods of NP fixation in the structure of the NFs is particularly important. The AATCC 61(2A)-1996 test method (simulating of washing), storage tests, and ion release tests are described in the literature as ways to confirm the durability of the antibacterial properties of modified NFs for water filtration [17,23,24,25]. However, these testing methods do not correspond to the real conditions of water filtration. We propose two efficient testing methodologies for the simulation of bacterial filtration under air and water filtration conditions.

The current study presents long-term and versatile research focused on the antibacterial modification of polyurethane (PU) NFs by particles of CuO for future filtration applications. The issue of electrospinning of composite solutions, the question of particle allocation inside of fibrous structures and a systematic approach to testing the durability of the antibacterial properties in the filtration area have all been investigated in detail. The present study was intended (1) to estimate the influence of two spinning electrodes on the ES of a PU solution with a modifier; (2) to select an appropriate particles size of CuO to ensure the stable fixation and avoid the aggregation of inorganic species inside the polymer solution and in the structure of future NFs; and (3) to prove the durable antibacterial properties of composite nanolayers under real conditions of water and air filtration. In the study, PU NFs were modified by nano- or microparticles (MPs) of CuO using the blending method prior to ES. Nanospider ES was provided by two spinning electrodes—a rotating cylinder with a needle surface and a static wire electrode, which is commercially used for the mass production of NFs. This was performed to determine the influence of the geometry of the spinning electrode on the spinnability of colloidal polymer solutions and on the structure of future composite layers. It was experimentally established that the rotating spinning electrode must be used for the fiber forming of efficient antibacterial filters. This electrode is required to prevent the precipitation of the particles of the modifier at the bottom of the bath with the polymer solution. As for the antibacterial properties, we found no apparent advantages of nanoparticles in relation to microparticles in terms of their antimicrobial efficiency for the filtration application of our samples. The reason for this is assumed to be the tendency of NPs to aggregate. Due to the formation of large aggregates, the nanoparticles lose their major advantage—a larger surface area in relation to their volume. Another significant input of the present study was the simulation of the antibacterial purification of air and water under real filtration conditions. This allowed us to verify that the produced polyurethane nanofibers modified by microparticles of CuO are suitable for durable usage and are safe for the environment.

## 2. Results

### 2.1. Influence of Micro- and Nanoparticles of CuO on the Properties of the PU Solution

#### 2.1.1. Viscosity

**Figure 1:**
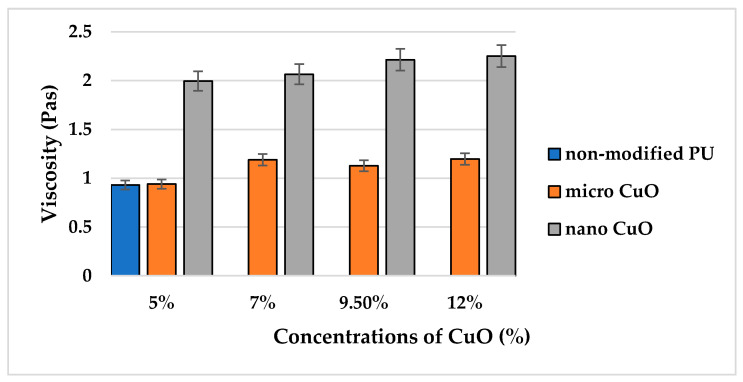
Comparison of average viscosities of the pristine polyurethane (PU) solution and solutions with micro- and nanoparticles of CuO.

The first step was to compare viscosities of the pristine PU solution and modified solutions with micro- (size distribution 700 nm–1 µm, average particle size 830 nm) and nanoparticles (average particle size 50 nm) of CuO. This is important because a major increase in viscosity may serve as the first indication of the solution not being suitable for processing by the electrospinning technique. There was a slight difference in the results of the viscosity measurement for the non-modified PU solution (first blue column in Figure 1) and solutions with microparticles of CuO, despite the comparatively high input concentrations of the micro-sized additive (orange columns in Figure 1). This indicates the homogeneous distribution of microparticles in the solution.

A comparison of the effect of micro- and nanoparticles on the rheological behavior of the polymer solution has a special significance in terms of the influence of these additives on the structural and dimensional characteristics of future nanofibers. It may indicate a higher tendency of the nano-sized additive to form aggregates in the polymer solution. One interesting fact was observed due to the measurement of the average viscosity of solutions with NPs. Despite the increase in nano-sized CuO concentrations from 5% to 12%, there was no significant difference in the values of the average viscosity for the nano-modified solutions. 

#### 2.1.2. Conductivity and Surface Tension

The declared value of electrical conductivity for CuO at room temperature is 640 µS/cm [25]. The results of measurements of conductivity and surface tension for the solutions with MPs and NPs of CuO are presented in Table 1 and Table 2, respectively.

In other studies, different salts are used to increase the conductivity of polyurethane solution for improvement of the spinnability. For example, in [26] tetraethylammonium bromide (TEAB) was added to polyurethane. Selected concentrations of TEAB 0–0.1–0.3–0.87–1.82% wt promoted a change of PU conductivity 91.5–460–1145–2970–5300 µS/m, respectively. Such a valuable increase in conductivity is explained by the chemical interaction between salt, the polymer and the solvent. In our research, the conductivity of modified solutions with both sizes of modifier increased insignificantly compared to the pristine PU solution. However, a clear dependence of the increase in conductivity with the growth of particle concentrations was not determined. Such behavior of the conductivity values of the modified solutions confirms that the used additives are chemically stable and do not react with the PU solution. If the used modifier had a chemical interaction with the polymer, then the conductivity of the composite solution would have increased considerably.

Another tendency was observed in the case of the surface tension measurement. The increase in particle concentrations (from 7%) led to a slight decrease in the surface tension compared to the pristine solution. This was expected, as the incorporation of particles violates the physical integrity of the polymer solution. However, the important conclusion is the fact that the introduction of modifiers into the PU solution had no significant effect on the change of values of conductivity and surface tension of the modified solutions. This proves the chemical stability of the used particles in the PU solution and indicates the possible spinnability of all of the modified solutions despite the considerable changes in the viscosity values of the solutions with NPs.

### 2.2. Structure of Produced Composite Nanofibers

Internal Morphology of PU Nanofibers with Micro- and Nanoparticles of CuO.

**Figure 2:**
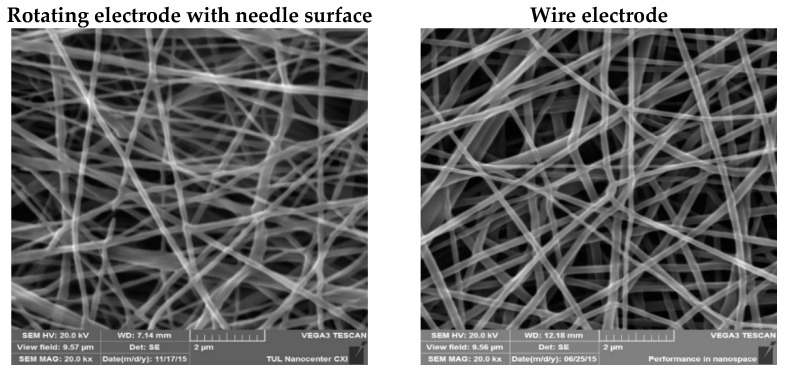
Scanning electron microscopy (SEM) images (20,000× magnification) of non-modified PU nanofibers produced from two different electrodes.

The scanning electron microscopy (SEM) analysis provided the internal morphology of the produced mats. Figure 2 demonstrates that there is no visible difference in the structure of the pristine PU nanofibers produced from the rotating needle cylinder and from the wire electrode.

**Figure 3:**
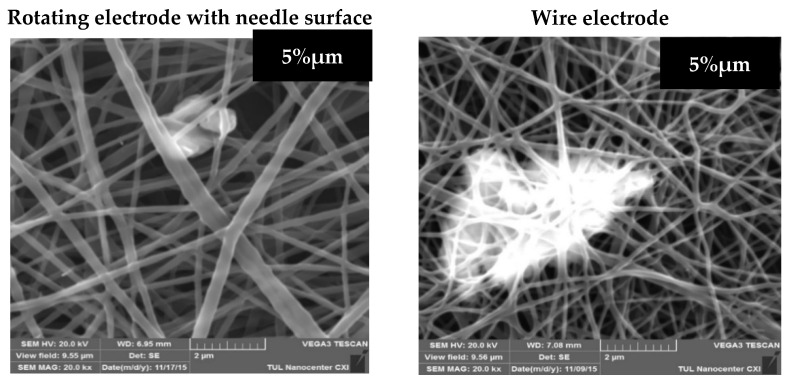

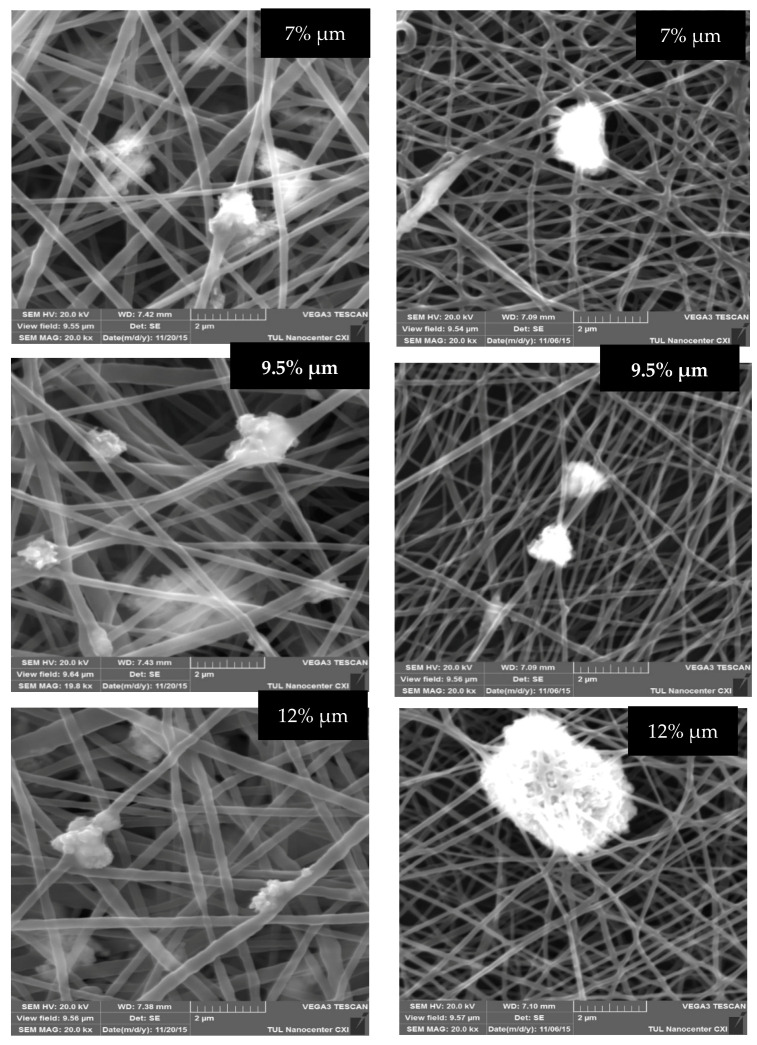
SEM images (20,000× magnification) of composite nanofibers produced from both electrodes with different concentrations of CuO microparticles.

Due to the analysis of the SEM images of the modified layers with a microscale additive, we are able to conclude that the samples with MPs of CuO produced from both electrodes (Figure 3) have a smooth structure with thin fiber diameters. However, there are large aggregates of micro-sized CuO at concentrations of 5% and 12% in the SEM images of samples produced from the wire electrode. Such large agglomerates of MPs in the PU mats were not observed in the images of layers produced from the needle cylinder. This may be explained by the fact that fiber forming is a long-term process, and the stirring of modified solutions is not ensured by the wire electrode, whereas the ES from the rotating electrode with the needle surface provides continuous mixing of the solution during the spinning process. The continuous mixing of the homogeneous PU solution without solid additives does not influence the production process, structure or properties of future nanofibers. But in this case the solutions were modified by particles of different sizes, so the prevention of the precipitation of the additives and the decrease in aggregation may significantly affect the ES process. Moreover, according to the SEM images, the microparticles of CuO are better distributed in the structure of polymer layers produced from the needle electrode.

**Figure 4:**
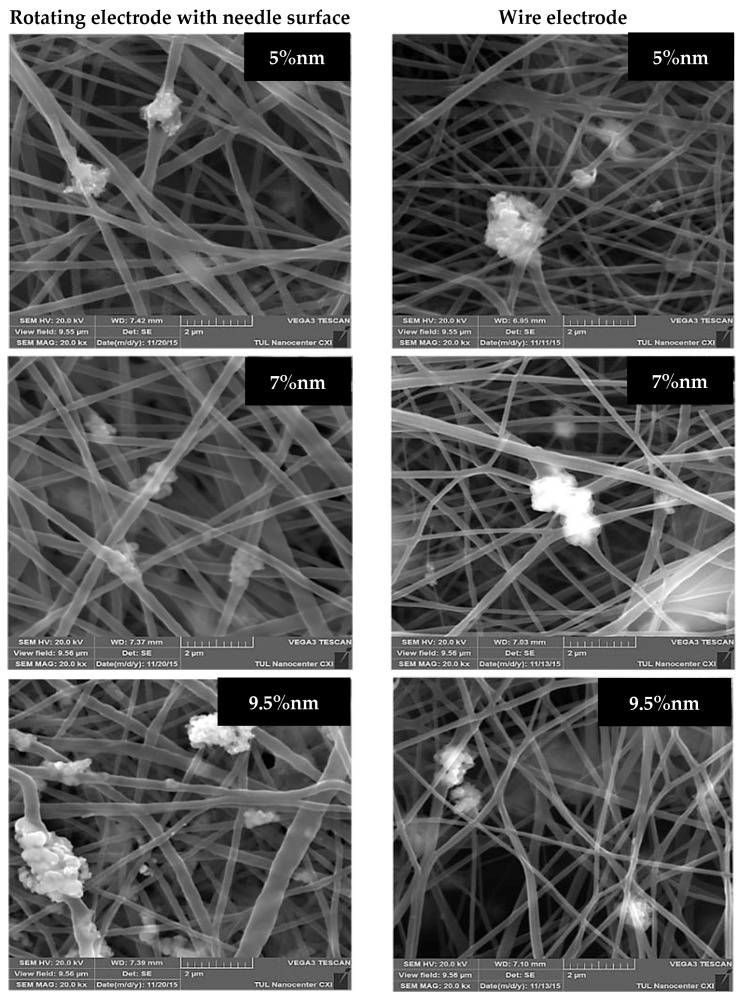

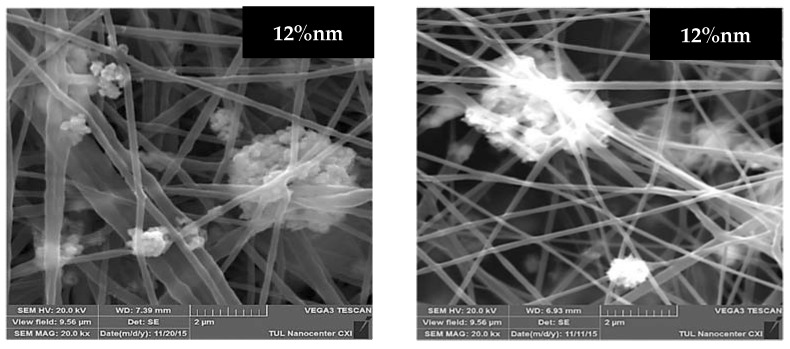
SEM images (20,000× magnification) of composite nanofibers produced from both electrodes with different concentrations of nanoparticles of CuO.

Large aggregates of NPs are visible in the structure of the produced substrates regardless of the electrode type (Figure 4). The distribution and size of the agglomerates of CuO NPs in the samples with lower concentrations (5% and 7%) of this additive produced from the rotating needle cylinder are more homogeneous than the same indexes for layers produced from the wire electrode. However, massive formations of nanoscale agglomerates are observed in samples with higher concentrations of antibacterial substance (9.5% and 12%) produced from both spinning electrodes. In our opinion, the continuous stirring provided by the rotating needle cylinder prevents the precipitation of NPs at the bottom of the bath with the solution but is unable to fully prevent the aggregation at high NP concentrations. The unique functional properties (including antibacterial) of nanoparticles are caused by their very small size, which provides them with an extensive surface area. Therefore, the formation of aggregates may be an obstacle to the manifestation of antibacterial properties of nanoparticles in full. In addition, there are doubts about the stability of such aggregates under further filtration application of the samples.

**Figure 5:**
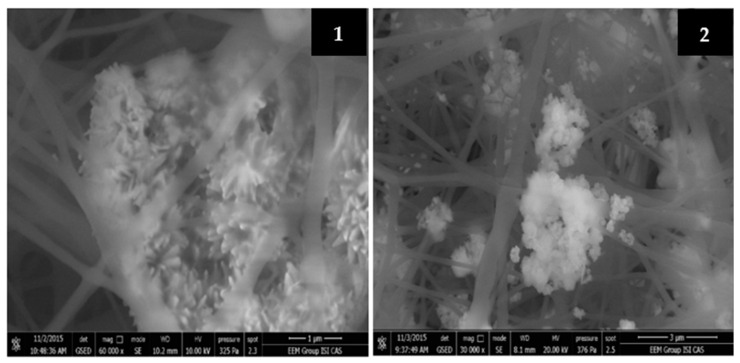
SEM images of PU nanofibres with 7% (**1**) and 12% (**2**) of CuO NPs produced from rotating electrode with a needle surface (60,000× magnification).

The tendency of NPs to aggregate was already mentioned many times so it is important to show and describe the structure of these aggregates. Figure 5 provides images of the fibrous samples of PU with 7% and 12% of CuO nm obtained by QUANTA 650FEG SEM. The figure clearly shows that the nanoparticles have formed very large aggregates containing dozens of nanoparticles connected in a single formation. This indicates an uneven distribution of nanoparticles in the fiber structure, which may lead to large losses of potential antibacterial “points” for the efficient contact with the bacteria. Another risk is that the nanoparticles may separate from the large aggregates by the stream of water or air during filtration and get into the environment. On the one hand, there is an example where the aggregation of NPs is protected by the nanofibrous web shown in Figure 5(1). But on the other hand, the opposite situation is shown in Figure 5(2) where there are large aggregates not covered by the fibers. So, the stability of such aggregates of NPs in the fibrous structure is unpredictable, and there is no way to control it.

**Figure 6:**
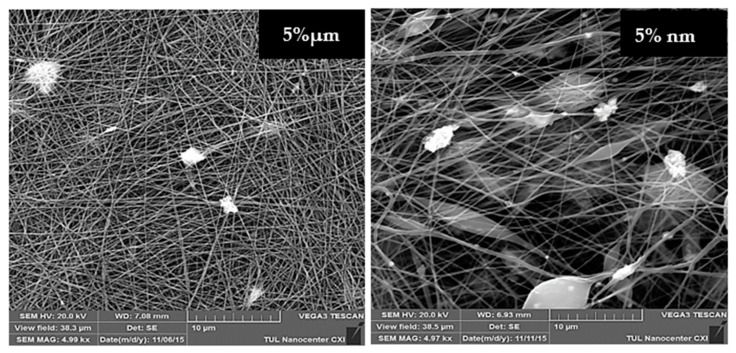
SEM images (5000× magnification) of nanofibres produced from the wire electrode with 5% of micro- and nanoparticles of CuO respectively.

It should be mentioned that the fibrous substrates with NPs produced from the wire electrode had many structural defects. Figure 6 provides SEM images of samples with 5% of micro- and nanoparticles of CuO produced from the wire electrode at a 5000× magnification. These images clearly show that the structure of the nano-modified sample contains many beads. It is possible that some part of the NP aggregates is located inside these beads. Regardless, the nanoparticles adversely affect the structure of the PU nanofibers produced from the wire electrode, which will negatively reflect on their filtration properties. The structures of all of the modified layers produced from the rotating electrode had no defects or beads.

Data on the modification of PU nanofibers with CuO microparticles are not available. In [17,18] the authors investigated the blending method for the introduction of CuO nanoparticles (CuO concentration range 1–10%) to a PU solution with further electrospinning using a laboratory device with a syringe connected to a high-voltage power supply. In both of these studies, layers with smooth fibrous surface and bead-free structure were observed. The presence of nanoparticles aggregates was not mentioned. In our study, an industrial electrospinning technology (Nanospider) was applied for the production of composite nanofibers. Such technology provides a continuous production cycle and produces a large amount of nanofibrous material. Respectively, the risk of aggregation of modifiers in the polymer solution is much higher than in the case of the syringe laboratory ES set-up with a shorter production cycle. However, it is worth mentioning that the aim of the presented research was to prove the possibility of the production of modified nanofibers for filtration application using an industrial ES method.

The diameter distribution of all of the produced layers is in the range of 75–650 nm. We observed a visible brown color, which became more intensive with increasing CuO concentrations. The average fiber diameters were calculated for each sample. By analyzing the results of the dimensional characteristics of the samples produced from the rotating needle cylinder, it is possible to state that the use of CuO led to an insignificant increase in the average diameters of the polyurethane nanofibers (Table 3). The CuO concentration of 7% had the most tangible impact on the increase in the diameters of the PU nanofibers, both for the micro- and nanoscale additives. The conductivity of CuO may have played a greater role when higher concentrations (9.5% and 12% wt) of the selected modifiers were added to the PU solution as it contributes to a thinning of the fibers. As shown in Table 3, the values of the fiber uniformity coefficients did not increase for the modified nanofibers compared to the pristine PU layer. This indicates a uniform distribution of diameters for all of the samples produced from the rotating roller, regardless of the dimensional characteristics of the modifiers.

Switching to the dimensional characteristics of nanofibers produced from the wire electrode, it is necessary to underline that the average diameters of the samples with MPs for the whole concentration range are smaller than this parameter for the pristine PU mat. The uniformity coefficients for micro-modified fibers do not differ or the values are very close to the non-modified substrate. The average diameters and uniformity coefficients of the composite layers with NPs are also similar to non-modified PU. Nevertheless, SEM analyses confirmed the presence of a large number of defects in the structure of samples with NPs produced from the wire electrode.

**Figure 7:**
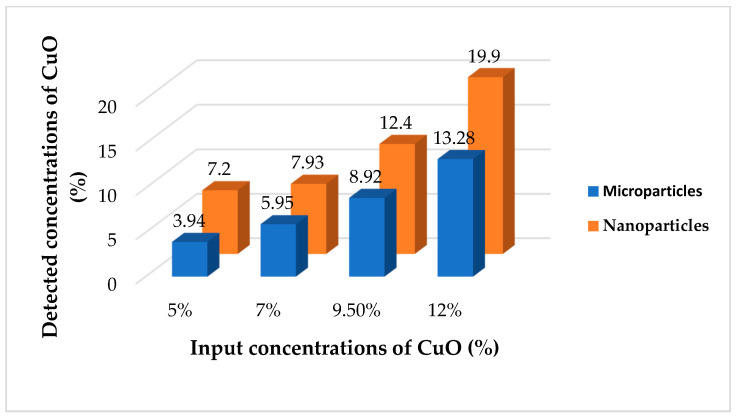
Difference between input and detected concentrations of micro- and nanoparticles of CuO for samples produced from the rotating electrode with needle surface provided by EDX analysis.

EDX analysis was performed to confirm the presence and approximate percentage content of micro- and nanoparticles of CuO in the structure of the nanofibers. In the case of samples produced from the needle cylinder, the detected amounts of CuO microparticles corresponded with the incorporated concentrations (Figure 7). At the same time, it was found that the detected concentrations of CuO nanoparticles for all of the samples were significantly higher than the introduced amounts of the modifiers (Figure 7). This may be explained by the tendency to aggregate and corresponds to the high values of viscosity of the PU solutions with CuO NPs. As nanoparticles possess a large surface area-to-volume ratio, the higher degrees of aggregation in the polymer solutions appear at lower concentrations of nano-sized additives. This is reflected in the uneven distribution of the nanoparticles on the surface of the fibers for the whole concentration range.

**Figure 8:**
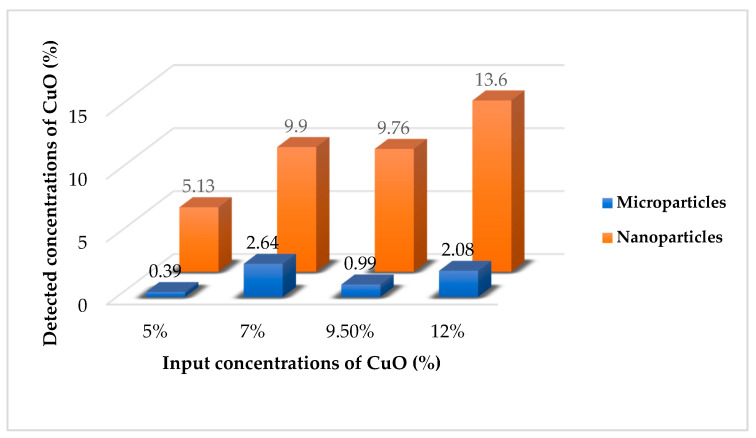
Difference between input and detected concentrations of micro- and nanoparticles of CuO for samples produced from the wire electrode provided by EDX analysis.

EDX analysis confirmed the presence of copper in the structures of all of the modified layers produced from the wire electrode (Figure 8). In the case of MPs, the detected concentration of CuO was much less than the incorporated amount of the modifier. This means that microparticles precipitated at the bottom of the bath with the solution and did not actively participate in the ES process together with the solution. The results of the EDX analysis confirmed our assumption that the rotating electrode with a needle surface is necessary for the efficient electrospinning of the colloidal solution of PU with microparticles of CuO. The detected concentrations of CuO NPs for samples with 5% and 9.5% correspond with the real amounts introduced to these solutions before ES. There is a difference between the measured and incorporated concentrations for the layers with 7% and 12% of nanoparticles. The detected amounts of NPs corresponded most precisely with the used concentrations (5%, 7%, 9.5% and 12%) for the samples with the nano-scale modifier. This may be explained by the fact that nanoparticles are less heavy in comparison with the micro-particles. Therefore, the nanosized CuO do not precipitate so quickly as the microparticles at the bottom with the polymer solution during ES using a wire electrode, which does not ensure mixing of the composite solutions.

The surface density of the prepared nanofibrous layers was calculated to compare the spinning performance depending on the concentration and size characteristics of CuO and the type of electrode (Table 4). The obtained data show that the micro- and nanoparticles contributed to an increase in the surface density of all of the modified nanofibers compared to the pristine PU mat produced from the needle cylinder. It is possible to observe (Table 4) that the smallest selected concentration (5%) of CuO MPs provides an five-fold improvement of this characteristic. Therefore, it is clear that the spinning performance of the polyurethane solution increased with the introduction of CuO. This effect is explained by the well-known conductive properties of copper. The influence of nanoparticles of CuO on the surface density of fibers produced from the rotating electrode is also positive but not as significant as the influence of the microparticles. The positive impact of nanosized CuO persists to the concentration of 7%. The index of the surface density of the sample with 12% of CuO NPs decreased and became approximately equal to the respective index of the nanofibrous layer with 5% of the modifier. Higher concentrations of nanoparticles (9.5% and 12%) led to the formation of larger amounts of aggregates (or larger sizes of aggregates), which may lead to a deterioration of the functional properties of the nanoparticles, including electrical conductivity. However, the key conclusion is that micro- and nanoparticles of CuO are not merely additives to impart antibacterial properties, but they also contribute to a significant increase in the electrospinning performance during the production of polyurethane nanofibers. Usually, even small concentrations of additives for the improvement of the production performance of the electrospinning process promote a substantial increase in the diameters of the fibers. However, this was not observed in the case of comparatively high concentrations (5–12%) of CuO. Therefore, the obtained results have important practical significance.

Measurements of surface density for samples produced from the wire electrode differ from Measurements of surface density for samples produced from the wire electrode differ from the respective results for samples from the electrode with a needle surface (Table 4). The positive tendency of the increase in surface density of the PU layers with incorporation of modifiers remained. However, the degree of influence of CuO on the performance of the ES process decreased for both sizes of CuO particles. This may also be explained by the difference in the construction of the spinning electrodes and in the contact conditions between the electrodes and the solution. In the case of the ES from the rotating electrode with a needle surface, the needle part of the electrode was immersed in the solution in the bath. In other words, the charged needles of the electrode were in continuous contact with the CuO in the modified solutions. The wire electrode (because it is thin and static) had a very short interaction with the solution in the bath.

### 2.3. Antibacterial Properties of Composite Nanofibrous Layers

It was observed that antibacterial activity increased with an increase in CuO concentrations for both sizes of particles and for both spinning electrodes (Table 5). There was no particular difference between the antibacterial properties of the samples with micro- and nanoparticles produced from the rotating needle cylinder. Therefore, it is possible to conclude that all of the composite layers, with the content of CuO particles in the concentration range of 7% to 12% produced from the needle electrode, demonstrated excellent antibacterial activity against the selected bacterial strains (gram-negative strain *Escherichia coli* (*E. coli*) and gram-positive *Staphylococcus gallinarum*). According to the results of the quantitative antimicrobial tests, the composite samples produced from the wire electrode had significantly lower activity against both of the tested bacterial strains compared to the layers produced from the electrode with a needle surface. Antibacterial efficiency against *Staphylococcus gallinarum* (*St. Gal*.) was particularly low. In fact, it is possible to conclude that such samples are not appropriate for the elimination of this strain (possibly for other gram-positive strains as well).

In the case of micro-modified samples produced from the wire electrode there are no questions about the reason of their lower antibacterial activity (Table 5). As was determined by EDX analysis (Figure 11) the concentration of CuO MPs in the structure of such PU nanofibers was much lower than the incorporated concentrations. Hence, poor antibacterial properties of the samples with MPs were expected. The results of the EDX measurement of NFs with a nanomodifier produced from the wire electrode were the opposite. However, as can be seen in Table 5, the samples with NPs did not demonstrate good antibacterial efficiency. There are two reasons for such results. The first reason is again the aggregation of nanoparticles, which was confirmed by the SEM images, and as described earlier, the aggregation led to a partial loss of the unique properties of the NPs. Many nano-scale particles are hidden inside clustered formations and they are not available for contact with bacteria due to aggregation. The second reason is also related to the formation of agglomerates, but in this case the aggregates that are not visible to the eye. There are many beads in the structure of the fibers with the NPs. If one assumes that these beads are filled with agglomerates of nano-scale CuO, then the low antibacterial activity of these samples may be easily explained.

**Figure 9:**
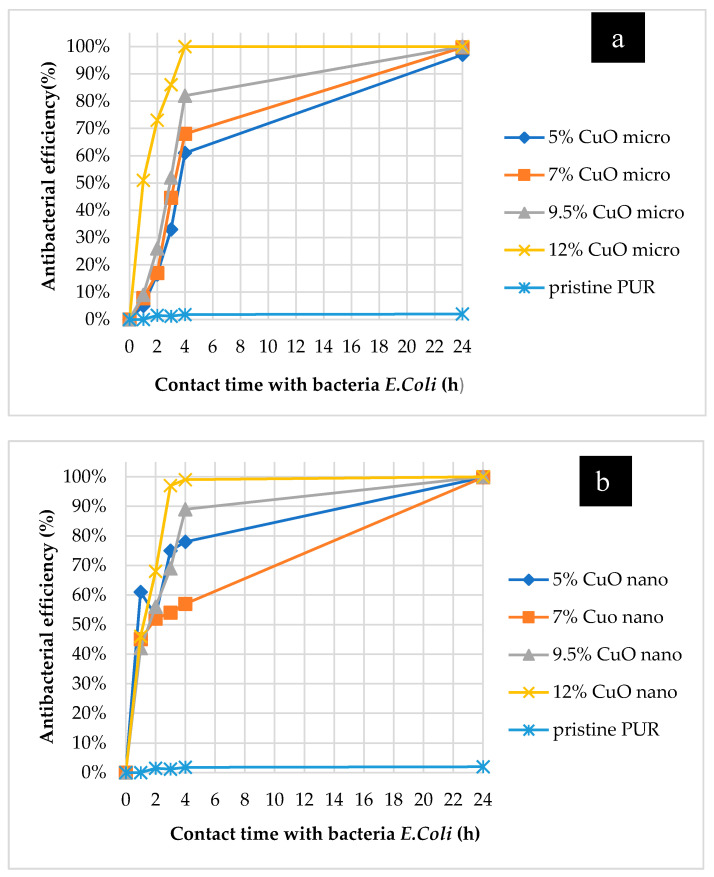
Changing of antibacterial efficiency against *E. coli* over time for samples with microparticles (**a**) and nanoparticles of CuO (**b**) produced from rotating electrode with a needle surface.

As it was confirmed that samples with both particle sizes produced form the rotating electrode demonstrated better antibacterial efficiency, then it is worth analyzing the manifestation of their activity over time. The change of antibacterial efficiency over time (from the minimum contact time between sample and bacteria (1 min) to the maximum contact time during the test (24 h)), is graphically represented in Figure 9. First of all, it is possible to conclude that the non-modified PU nanofibers did not exhibit activity after a prolonged contact time, which means that the selected polymeric material without proper modification is inert to bacteria. The second important conclusion is that the nanoparticles began to exhibit their antibacterial efficiency faster (after 1 h of contact—Figure 9b) than the microparticles (after 4 h, Figure 9a). However, in terms of filtration application, there is no fundamental difference if the captured bacteria begin to perish within 1 or 4 h after hitting the surface of the filter. Nevertheless, it is important that the antimicrobial properties of the modified layers produced from the rotating electrode with both particle sizes are almost identical after 24-h contact between the bacteria and the samples (Table 5, Figure 9).

### 2.4. Antibacterial Air Filtration Efficiency

There are only a few approaches to the measurement of the bacterial filtration efficiency of nanofibers in the literature. Polyacrylonitrile-Ag (PAN-Ag) composite nanofibers were investigated for the filtration of microorganisms and dust particles. The filtration testing apparatus consisted of a glass chamber, a sterile dish with nutrient solution and an outlet with a vacuum at the bottom of the device. The glass chamber was closed by a layer of PAN-Ag nanofibers. Ambient air passed through the composite filters and the penetrated microorganisms were caught on the surface of the nutrient solution. This testing method is indicative of bacterial filtration properties, but it is impossible to provide a quantitative estimation of bacterial filtration efficiency using such a method [27]. Polyvinylidene fluoride-Ag (PVDF-Ag) nanofibers showed 99.86% of bacterial filtration efficiency according to ASTM F 2101 with *Staphylococcus aureus* (*S. aureus*) aerosol. BFE of modified PVDF samples was measured in an Andersen sampler [28]. In the presented study, the measurement of bacterial filtration properties was examined by the AMFIT 13 method. This method was officially certificated by the Czech Environment Management Center. BFE was evaluated by a modified ASTM F2101-01.2001 methodology (test method for evaluating the bacterial filtration efficiency of medical mask materials using biological aerosol). The rates of air flow through the filter correspond with standards EN 1822 and EN 779 intended for filtration within the ventilation of buildings, as well as standards EN 143, EN 149 and others intended for tests of respirators and personal protective equipment.

Our results from the bacterial filtration test correspond with values of the surface density for all of the prepared samples. As mentioned above, the surface density of the nanofibers modified by MPs and produced from the needle cylinder was higher than the layers containing NPs of CuO. The samples with 7% 9.5% and 12% of microsized CuO demonstrated the highest surface density values. These samples showed a 100% bacterial filtration efficiency.

Such results lead us to the assumption that it is sufficient to use nanofibers with high surface density for bacterial air filtration and it is not necessary to pay attention to the antibacterial modification of nanolayers. However, this assumption is erroneous. The capture of bacteria on the surface of filter is only the first task to be solved. The second important objective is to eliminate the trapped bacteria for which antibacterial agents play a key role. Table 6 shows that the results of a “smear-test” confirmed the antibacterial activity of all of the modified nanofibers produced from the rotating electrode in eliminating the captured bacteria after the bacterial filtration test. The samples with 9.5 and 12% of MPs demonstrated the most remarkable results, with the complete elimination of trapped bacteria.

The bacterial filtration efficiency of the composite samples with MPs produced from a wire electrode was better than with the pristine PU layer or layers with NPs (Table 7). The surface densities of the micro-modified substrates were higher; therefore, they played a key role in the capability of the samples to capture bacteria. Moreover, the structure of the samples with nanoparticles was damaged by the presence of large numbers of beads. The results of the smear-test are also in favor of microparticles. However, none of the filters produced from the wire electrode are appropriate for the complete removal of bacteria trapped on the filtering medium. The lower elimination abilities of nano-modified mats to remove captured bacteria confirmed that a considerable part of the NPs is hidden inside the aggregates, fibers and beads.

### 2.5. Stability of Antibacterial Properties of Modified Nanofibers

This part of our study has special significance. Previous research [18] focusing on the antibacterial modification of PU nanofibers by CuO provides no data on the stability of CuO fixation in the fibrous structure and durability of antimicrobial efficiency.

In the presented research it was decided to continue with the measurement of the antibacterial stability and fixation of CuO only for modified samples produced from the rotating needle electrode. This decision was made based on the results of the bacterial air filtration tests.

**Figure 10:**
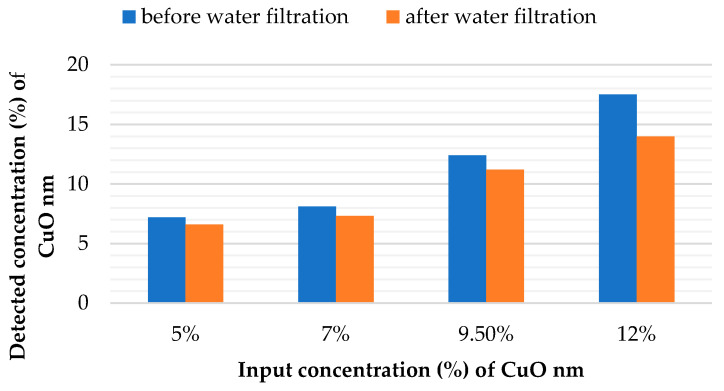
Content of nanoparticles of CuO on the nanofiber surface before and after treatment under the simulated conditions of water filtration according to the results of EDX analysis (experiments with the rotating needle electrode).

A very important aim of this research was to confirm that CuO particles were securely fixed into the structure of the nanofibrous matrix. Therefore, each sample was treated under the simulated conditions of water filtration. EDX analysis was repeated to compare the number of antibacterial additives on the surface of the fibers before and after the water treatment test. No difference was found by comparing the results of the EDX analysis of the composite samples with the microparticles produced from the rotating electrode before and after the water filtration test (Figure S1 and Table S1). The results for samples with nanoparticles showed a different tendency. Figure 10 shows that a number of CuO nanoparticles were poorly fixed into the structure of the produced layers. It was established that the nanoparticles formed sufficiently large aggregates in the fibrous structure. Some of the nanoparticles in the structure of these aggregates were not immobilized inside of polymer matrix and may have been connected to neighboring particles only by physical interaction. This may explain the observed tendency of the nano-sized additive to wash out. Nevertheless, the results of the EDX analysis from the standpoint of the percentage ratio of the detected compounds are only orientational. The results of antibacterial tests of samples used for water filtration would provide a more demonstrative criterion for particle fixation. For this purpose, after testing under simulated conditions of water filtration, the nanofibers with micro- and nanoparticles according to the Cornell test to determine their efficiency against *E.coli* and *St. Gal*.

No change in antibacterial efficiency was detected in the case of nanofibers modified by microparticles and produced by the rotating electrode. The results for samples with the nanomodifier are slightly different. Table 8 shows that a decrease in antibacterial activity was found for the composite mats within the whole concentration range of nanoparticles against both strains. The change in antibacterial properties after the water filtration test is a very important criterion for the selection of an appropriate material for application of water filtration. Only the samples with stable and long-term antibacterial activity may be presented as suitable for water purification, and the results of the antibacterial and filtration investigations showed that PU nanofibers with CuO nanoparticles did not fulfill these requirements. The release of NPs into the environment is potentially dangerous and should be strongly controlled even on the experimental level.

## 3. Discussion

There are many scientific studies about the production of nanofibers modified by nanoparticles of metal oxides produced from the syringe laboratory set-up [23,24,28]. This type of production involves a short-term process, so the aggregation of NPs in the fibrous structure is not observed due to the very limited time of fiber forming. However, our aim was to produce composite antibacterial nanofibers by insertion of a modifier into the polymer solution using industrial production technology and to study the properties and structure of the produced layers. Therefore, we did not use the syringe laboratory set-up in our particular research. Production of modified nanofibers with particles of metal oxides using the Nanospider electrospinning technique has not been extensively studied. For this reason, it is very difficult to compare our results with the conclusions of other studies.

Only a few studies have presented the results of the modification of polyurethane nanofibers by nanoparticles of CuO in order to impart their antibacterial and conductive properties [17,18]. In the mentioned studies, modified layers were produced using the syringe laboratory set-up, and, as it could be predicted, the functional properties of modified nanofibrous substrates increased with an increase in nanoparticle concentrations. However, the antimicrobial activity of modified layers under filtration conditions, as well as the influence of CuO on the electrospinning and structure of fibers, was not subject to intensive consideration.

Our study has special scientific meaning due to its complex approach to the problem of nanofiber modification and the future filtration application of composite substrates. Both micro- and nanoparticles of CuO were encapsulated into the structure of PU mats. Moreover, the influence of two spinning electrodes on the functional properties of CuO and on the allocation of modifiers in the structure of composite nanofibers was investigated in detail.

The antibacterial mechanisms of NPs depend on composition, surface modification, intrinsic properties and the bacterial species [29]. Several studies have indicated that the interaction of nanoparticles with a bacterial cell occurs in stages. In the first (physical) stage, the metal nanoparticles are adsorbed on the surface of the microorganism due to the resultant electrostatic pressure. After that, nanoparticles get inside. This is confirmed by submicroscopical studies. In the following stages (molecular and cellular), the cellular membrane is changed, with emboly, perforation and enlargement of the cellular wall taking place. The perforation of the cellular wall of a microorganism by nanoparticles leads to the discharge of the intracellular matrix [20]. It was found that CuO nanoparticles exhibit antibacterial activity to gram-negative (*E. coli*) and gram-positive strains (*S. aureus*) [30]. In bacteria, the Gram strain shows an important classification system, where several cell properties may be correlated with the cell envelope. Gram positive bacteria have a thick (20–80 nm) cell wall as the outer shell of the cell. This is contrasted with Gram negative bacteria, which possess a relatively thin (<10 nm) cell wall layer but harbor an additional outer membrane with several pores and appendices. These differences in the cell explain the differing properties, in particular their responses to heat, UV radiation and antibiotics [31]. CuO nanoparticles are able to attach to the bacterial cell and to engender reactive oxygen species (ROS), which promotes the intracellular oxidative stress for both gram-negative and gram-positive strains [30]. In the presented study, it was confirmed that microparticles of CuO are very efficient antimicrobial agents against both gram-negative and gram-positive strains.

The results of measurements of the bacterial air filtration efficiency and smear-tests demonstrated that the use of a wire electrode is not suitable for the production of composite antibacterial nanofibers from modified PU solutions. The absence of continuous stirring of the colloidal solutions leads to the aggregation of both dimensional types of modifier, to the precipitation of MPs at the bottom of bath with polymer solution, and to the formation of agglomerates of NPs inside polymer beads. Nanofibers with CuO microparticles produced by a needle rotating electrode proved to be more efficient and stable for bacterial air purification. Micro-modified nanofibrous layers are able to capture and eliminate more bacterial units due to their higher surface density, better distribution and availability of CuO on the surface of the samples.

The storage test is used in the literature to confirm long-term antibacterial properties. For example, CA, PAN and PVC nanofibers with AgNO_3_ were stored in a refrigerator for six months. The antibacterial properties of modified nanofibers were the same both before and after storage [25]. This type of test may characterize the preservation of antimicrobial activity over time and may be used for materials for medical applications. Filtration samples require a different testing approach. Durable antibacterial PAN-Ag nanofibers were produced by ES. A silver ion release test was performed using atomic absorption spectroscopy to confirm the stability of antibacterial modification [24]. In our opinion, the ion release test is suitable for materials for biomedical applications.

There are interesting data from the determination of the stability of the fixation of NPs of MgO and single-walled carbon nanotubes in the structure of PAN nanofibers for air filtration applications. In this study, air samples were collected downstream of prepared modified nanofibers using a mixed cellulose ester (MCE) filter according to the National Institute for Occupational Safety and Health (NIOSH) method 7402. Then, MCE filters were investigated by CytoViva’s Hyper Spectral Imaging System coupled with Enhanced Dark Field Microscopy (HIS-EDFM) for assessing the potential release of nanomaterials (carbon nanotubes and MgO NPs) from composite nanofibrous layers [32]. This method is well-suited to air filters only. Water filtration conditions are more aggressive. On the one hand, there are many studies on antibacterial nanofibers for water filtration applications, but their durable bactericidal efficiency has not been deeply studied [6]. For this reason, our study focused on the stability of the antibacterial modification of PU nanofibers with CuO particles investigated by the testing methodology under simulated conditions of water filtration. 

Nevertheless, the deterioration of the antibacterial properties of samples modified by nanoparticles after the water filtration test allowed us to state that a certain amount of the nanomodifier is poorly fixed and washes out from the nanofibrous layers. This finding is extremely important because the release of nanomaterials into the environment must be kept under strict control. Confirmation of the stability of nanoparticles in the structure of filters is only one way of verifying the possibility to use them for air or water filtration. Therefore, our study shows that nanoparticles of CuO are not appropriate and not safe for selected applications. However, the incorporation of microparticles of CuO to the PU solution with further electrospinning from the rotating electrode with a needle surface is a perspective and, most importantly, stable technology for the production of nanofibrous filters for air and water filtration membranes.

## 4. Materials and Methods

### 4.1. Materials

In this work, polyurethane (Larithane LS 1086, aliphatic elastomer based on 2000 g/mol, linear polycarbonated diol, isophorone diisocyanate and extended isophorone diamine) was used as a polymer. Larithane LS 1086 was dissolved in dimethylformamide. The polyurethane was obtained from Novotex (Gaggiano MI, Italy). Dimethylformamide (DMF) and microparticles of copper oxide with a size distribution of 700 nm^−1^ µm were purchased from Penta (Prague, Czech Republic). We also used nanoparticles of CuO with an average diameter of 50 nm purchased from Sigma Aldrich (St. Louis, MO, USA). Gram-negative (*Escherichia coli*) and gram-positive (*Staphylococcus gallinarum*) strains were utilized as model organisms to check the antimicrobial properties of the produced nanofibers. The bacteria were obtained from the Czech Collection of Microorganisms (Masaryk University in Brno, Brno, Czech Republic). The nutrient medium Tryptone Soya Broth (TSB) and sterile Tryptone Soya Agar (TSA) from Oxoid CZ s.r.o. (Brno-Tuřany, Czech Republic) were used for the inoculation and the incubation of bacteria.

The PU solution was prepared at a 15% concentration in DMF. Then, micro- or nanoparticles of CuO were added to obtain colloidal solutions with different concentrations of antibacterial agents (5%; 7%; 9.5%; 12 wt%). These composite systems were mixed using magnetic stirrers for 12 h prior to ES.

### 4.2. Properties of the Solution

The measurements of viscosity, surface tension and electrical conductivity helped to determine the influence of different dimensions and concentrations of the CuO particles on the properties of the PU solution. Rheological properties were measured using a Rheometer HAAKE Roto Visco 1 (Thermo Fisher Scientific, Waltham, MA, USA) at 23 °C. The measuring part of the device consists of a rotary disc and stationary plate with a working gap of 1.45 mm. A drop of the polymer solution is deposited on the stationary plate. The rotary disc is immersed into the solution. The required force to overcome the resistance to rotation is measured during the disk’s rotation. The measured data were processed by the software Haake RheoWin^®^ (Thermo Fisher Scientific, Waltham, MA, USA), which consists of two units. The surface tension of pristine and modified solutions was measured by the bubble pressure method. The measurement was provided by a portable bubble tensiometer PocketDyne (KRUSS Scientific Instruments, Matthews, NC, USA). The conductivity was investigated using a CyberScan CON 510 conductivity meter (EUTECH instruments, Singapore).

### 4.3. Electrospinning Process—Used Spinning Electrodes

The ability to produce filtration materials on an industrial scale is very important. In fact, the real practical application of some materials is not feasible if their production in the required quantity has not been proven using an affordable and implemented technology. Therefore, we produced composite polyurethane nanofibers using the industrial Nanospider technique. In the present study, two types of spinning electrodes were chosen and compared.

#### 4.3.1. The Roller Spinning Method

**Figure 11:**
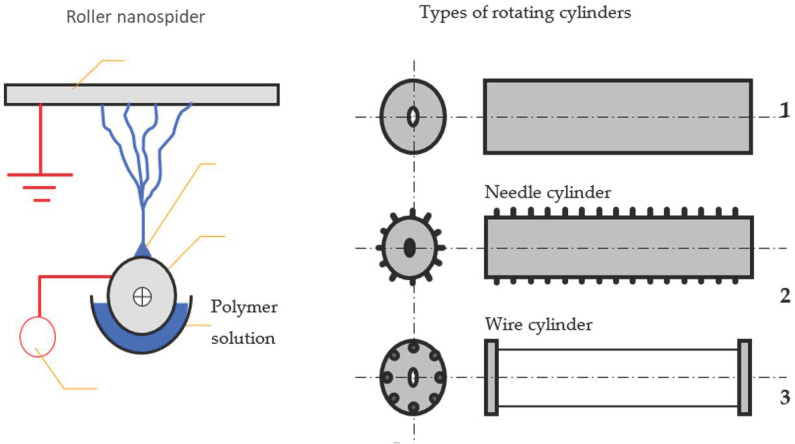
Diagram of the Nanospider method with different types of spinning electrodes (1—smooth cylinder, 2—needle cylinder, 3—wire cylinder).

The Nanospider (Figure 11) consists of a rotating cylinder (spinning electrode) to spin fibers directly from the polymer solution. The cylindrical rotary electrode with a needle surface was used as the first technique for the fiber forming process. This type of electrode was chosen in order to ensure the stirring of the colloidal solution and to prevent particles from aggregating and being deposited at the bottom of the dish with the PU. In the present study, the spinning solution was filled into a polypropylene dish. The electrode with a needle surface was partially immersed into the polymer solution and, as it rotated, a controlled amount of the polymer solution was carried to the top parts of the needles on the surface of the cylinder in the electric field where a series of Taylor cones was created.

A high voltage was connected to the rotating roller. The high voltage induced the necessary charges on the solution and together with the external electric field, initiated the ES process when the electrostatic force overcame the surface tension of the solution. As the solvent evaporated, the jets of the polymer solution were transformed, and solid nanofibers were obtained before reaching the collector electrode. The nanofibers were collected on the polypropylene spun bond nonwoven antistatic material. The advantages of this method are the continuity of the process and a large productive capacity [29,30]. All of the parameters of the process of electrospinning the PU modified solutions were determined experimentally, and are presented below:

- voltage = 67 kV
- roller speed = 2.5 rpm
- speed of collecting material = 0.05 m/min
- distance between the rotating cylinder and collector electrode = 16 cm
- temperature (T °C) = 20 °C
- humidity in the spinning chamber = 22%.

#### 4.3.2. Wire Spinning Electrode

The second electrode used to produce the PU nanofibers with antibacterial additives was a static wire electrode. Electrospinning was performed by the Nanospider laboratory machine NS LAB 500S (from Elmarco s.r.o., Liberec, Czech Republic) with an air conditioning unit. The optimum spinning parameters for the production of modified PU solutions from the wire electrode were determined experimentally, and are presented below:

- voltage = 60 kV
- traversing speed of wire = 0.2 mm/s; speed of collecting material = 0.05 m/min
- distance between the wire and collector electrode = 17.5 cm
- temperature (T °C) = 20 °C
- humidity in the spinning chamber = 22%.

This technique does not provide continuous stirring of the solution during the electrospinning process in contrast to the rotating electrode with a needle surface. Nevertheless, this type of electrode is most commonly used for the mass production of NFs. Therefore, it is possible to expect the necessity to stir the modified solutions during the fiber forming process and to select a more appropriate spinning surface for the processing of composite nanofibers.

### 4.4. Structure of Produced Materials

The morphology and elemental composition of all of the produced nanofibrous layers was analyzed using TESCAN VEGA3 SEM and Carl Zeiss ULTRA Plus scanning electron microscopes with an OXFORD Instruments (Abingdon-on-Thames, Great Britain) microanalytical system equipped with an energy dispersive X-ray spectrometer (EDX). Some of the samples were studied by QUANTA 650FEG (FEI Company, Hillsboro, OR, USA) scanning electron microscope at the Department of Environmental Electron Microscopy of the Institute of Scientific Instruments of the Czech Academy of Sciences. The average diameter of the fibers and the net diameter distribution of the samples with different CuO concentrations were measured and calculated from SEM images using Lucie 32G computer software. The fiber uniformity coefficient was determined using number and weight average calculations. The number average is known as an arithmetic mean in mathematics. The method for calculating the uniformity coefficient has the same principle as the molar mass distribution in chemistry. We calculated both of these values using Equations (1) (*A_n_*—number average or average diameter) and (2) (*A_w_*—weight average), which are given below:

(1) $$A_{n}=\frac{\sum n_{i}d_{i}}{\sum n_{i}}$$

(2) $$A_{w}=\frac{\sum n_{i}d_{i}^{2}}{\sum n_{i}d_{i}},$$

where *d_i_*—fiber diameter; *n_i_*—fiber number. The fiber uniformity coefficient was determined by the *A_w_*/*A_n_* and the optimum value should be very close to 1 for fibers with a uniform diameter distribution [26]. EDX analyses of all of the produced nanofibers were performed with the purpose of assessing the presence of CuO and making indicative conclusions about the concentration of the modifier on the surface of the samples. Moreover, the surface density of the prepared samples was calculated to compare the influence of additives on the spinning performance of the polyurethane solution.

### 4.5. Antibacterial Properties of Composite Nanofibrous Layers

Standard Test Method ASTM E2149^1^ was used to determine the antibacterial efficiency of the produced samples. The test quantitatively evaluates the efficiency of materials treated with antimicrobial agents (fabrics, textiles with non-leaching additives, paper, granular materials, ceramics, plastics, glasses, stoneware) under dynamic contact conditions between the tested samples and the bacterial suspension. The antimicrobial activity of the nanofibers was tested against gram-negative *Escherichia coli* and gram-positive *Staphylococcus gallinarum* bacterial strains. Changes in the antibacterial activity of each of the produced nanofibrous layer were monitored over time (from 1 min to 24 h). The results are expressed as a percentage (%) of reduction (CFU/mL) after a defined duration of contact between our sample and the bacterial suspension (0 min; 60, 120, 180, 240 min; 24 h).

### 4.6. Stability of Particles Fixation into the Nanofibrous Structure

All of the produced samples were tested under simulated conditions of water filtration. It was decided to determine the stability of particle fixation based on the results of the water filtration test because its conditions are more aggressive than air filtration and the probability of the bed-fixed particles washing out is higher. Water passed through each sample at the flow rate of 180 L/h for 8 h (1440 L through each sample—this amount of water corresponds to a two-week water consumption per person per household in the Czech Republic). Quantitative antibacterial tests and EDX analyses of all of the treated samples were repeated after the water filtration test in order to compare the results of these two tests before and after water filtration and to verify the stability of the antibacterial properties and fixation of CuO particles in the structure of the nanofibers.

### 4.7. Measurement of Bacterial Filtration Efficiency

**Figure 12:**
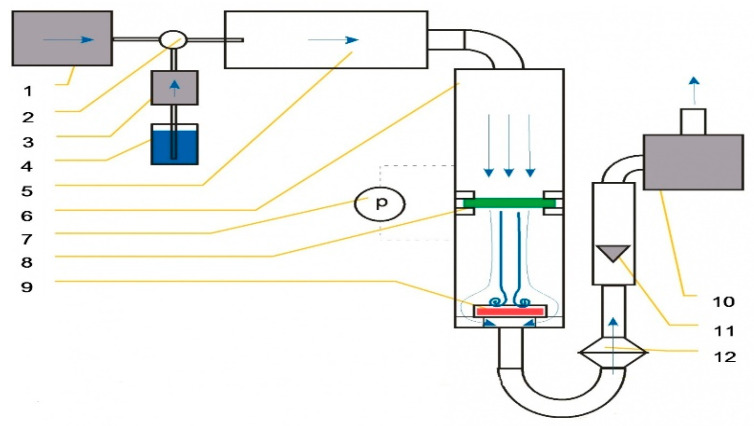
Diagram of AMFIT-13: 1-source of compressed air; 2—atomizer, 3—peristaltic pump (dosage control); 4 -reservoir with inoculum; 5—tube for atomizing; 6—stabilizing tube with direction of air and aerosol flow in front and behind the sample; 7—pressure gauge sensors; 8—tested filter; 9—Petri dish with nutrient agar; 10—vacuum pump; 11—float rotameter; 12—HEPA filter (for capturing bacterial aerosol passing through the tested filter).

This part of the experiment is particularly important from the point of view of an evaluation of the practical application of our samples under real conditions of bacterial air filtration. The bacterial filtration efficiency of pristine and modified nanofibers was tested using a special AMFIT 13 device (Anti-Microbial Filtration Tester). The AMFIT 13 devise (Figure 12) was applied to verify the extent to which the filter is able to prevent penetration of aerosolized inoculum with the bacteria to the purifying area. The method does not determine whether this objective has been achieved by the mechanical capture of bacteria on the filter or by their inhibition due to antibacterial modification of nanofibrous filtration materials. The essence of this measurement is a simulation of a passage of aerosolized contaminated inoculum through the tested sample. The presence of bacteria, which are injected into the testing apparatus and which pass through the filter media, has been analyzed. Petri dishes with agar were used to determine the number of bacteria in the device. They were placed at the end of the apparatus. Bacteria were captured on the surface of the agars and detected after incubation (for 24 h at 37 °C). Bacterial filtration efficiency (%BFE) is defined similarly to particulate filtration according to Equation (3):

(3) $$\%BFE=\left(1\;-\frac{n_{1}}{n_{2}}\right)\times 100,$$

where $n_{1}$—the number of colonies on the agar surface when the Petri dish is placed behind the tested filter (i.e., the amount of bacteria that have not been captured by the filter); $n_{2}$—the number of colonies on the agar surface without the presence of the filter (i.e., the real amount of bacteria that have been introduced into the testing apparatus).

When the bacterial filtration efficiency was confirmed, it was necessary to assess the ability of the filters to remove the captured bacteria (hereinafter a “smear test”). The smear test was performed in accordance with the procedure described below. One mL of nutrient medium was inoculated on the surface of a new agar. The sample with the bacteria captured after the bacterial filtration test was placed on this agar with the medium. The agar plate with a filter was placed in the incubator with a mechanical rotator (for uniform distribution of nutrient medium on the agar surface) for 8 h at 37 °C. An eight-hour time period was experimentally set as the time required for the CuO to manifest its antibacterial properties in full. Then, the sample was removed by sterile pincers from the agar surface. As the last step, this agar was incubated for another 16 h at 37 °C. The total incubation time was 24 h. Finally, the number of grown colonies was counted.

## Figures and Tables

**Table 1:** Values of conductivity and surface tension for solutions with microparticles of CuO.

Solution Properties	0%	5%	7%	9.5%	12%
Conductivity (µS/cm)	436	440	450	448	452
Surface tension (mN/m)	70.5	71.1	68.4	68.1	66.7

**Table 2:** Values of conductivity and surface tension for solutions with nanoparticles of CuO.

Solution Properties	0%	5%	7%	9.5%	12%
Conductivity (µS/cm)	436	447	452	453	451
Surface tension (mN/m)	70.5	70.9	66.2	67.8	67.2

**Table 3:** Results of the measurement of fiber diameters and calculation of uniformity coefficients.

Sample Rotating Needle Cylinder	$A_{n}\;\mathrm{nm}$	95% Confidence	$A_{w}\;\mathrm{nm}$	K Aw/An	Sample Wire Electrode	$A_{n}\;\mathrm{nm}$	95% Confidence	$A_{w}\;\mathrm{nm}$	K Aw/An
Pristine PU	182	5.4	194.5	1.07	Pristine PU	189	5.9	199	1.05
PU + 5%CuO µm	226	6.2	239	1.06	PU + 5%CuO µm	134	4.04	142	1.06
PU + 5%CuO nm	228	6.04	239	1.05	PU + 5%CuO nm	188	6.15	202	1.07
PU + 7%CuO µm	278	8.5	298	1.07	PU + 7%CuO µm	142	3.5	149	1.05
PU + 7%CuO nm	262	5.97	270	1.03	PU + 7%CuO nm	175	5.4	186	1.06
PU + 9.5%CuO µm	242	6.9	257	1.06	PU + 9.5%CuO µm	125	3.2	132	1.06
PU + 9.5%CuO nm	237	6.1	249	1.05	PU + 9.5%CuO nm	184	6.3	198	1.08
PU + 12%CuO µm	231	5.7	249	1.08	PU + 12%CuO µm	139	3.3	145	1.04
PU + 12%CuO nm	226	6.9	240	1.06	PU + 12%CuO nm	181	6.1	195	1.08

**Table 4:** Results of measurements of the surface density for nanofibrous layers with micro- and nanoscale CuO produced from the rotating needle electrode and wire electrode.

Sample	Surface Density of Produced Nanofibers (g/m²)	
	Rotating Electrode with Needle Surface	Thin Wire Electrode
Pristine PU	2.5	3.43
PU + 5% CuO µm	12.28	4.88
PU + 5% CuO nm	4.56	3.11
PU + 7% CuO µm	13.05	5.31
PU + 7% CuO nm	9.89	4.48
PU + 9.5% CuO µm	13.93	7.63
PU + 9.5% CuO nm	7.41	6.57
PU + 12% CuO µm	19.46	7.25
PU + 12% CuO nm	5.38	6.18

**Table 5:** Antibacterial efficiency of composite nanofibers (NFs) with micro- and nanoparticles produced from both electrodes against *Escherichia coli* and *Staphylococcus gallinarum* (contact time between bacterial solutions and modified samples 24 h).

Sample	Efficiency (%)—*Escherichia coli*		Efficiency (%)—*Staphylococcus gallinarum*	
	µm	nm	µm	nm
PU + 5% CuO				
cylinder	97	96.8	98.8	62.7
wire	64	85	0	17
PU + 7% CuO				
cylinder	99.7	99.8	100	96.2
wire	67	90	23	20
PU + 9.5% CuO				
cylinder	100	100	100	98.8
wire	70	89	29	16
PU + 12% CuO				
cylinder	100	100	100	99.6
wire	81	96	30	30

**Table 6:** Results of the bacterial filtration test and “smear-test” for samples produced from the rotating electrode with a needle surface.

Sample	Number of Bacteria Passed through the Sample	BFE (%)	Number of Survived Bacteria after the “Smear-Test”
Inoculum	320	-	-
PU pristine	17	95	278
PU + 5% CuO µm	5	98	6
PU + 5% CuO nm	15	95	13
PU + 7% CuO µm	0	100	3
PU + 7% CuO nm	9	97	45
PU + 9.5% CuO µm	0	100	0
PU + 9.5% CuO nm	11	96.6	30
PU + 12% CuO µm	0	100	0
PU + 12% CuO nm	11	96.6	19

**Table 7:** Results of the bacterial filtration test and “smear-test” for samples produced from the wire electrode.

Sample	Number of Bacteria Passed through the Sample	BFE (%)	Number of Survived Bacteria after the “Smear-Test”
Inoculum	312	-	-
PU pristine	23	93	303
PU + 5% CuO µm	11	96	14
PU + 5% CuO nm	13	95.8	52
PU + 7% CuO µm	0	100	2
PU + 7% CuO nm	30	90	23
PU + 9.5% CuO µm	1	99.7	7
PU + 9.5% CuO nm	26	92	22
PU + 12% CuO µm	0	100	25
PU + 12% CuO nm	75	76	43

**Table 8:** Differences in antibacterial activity of samples with nanoparticles of CuO before and after treatment under the simulated conditions of water filtration (experiments with the electrode with a needle surface).

Tested Sample	Efficiency (%)— *Escherichia coli*		Efficiency (%)— *Staphylococcus gallinarum*	
	Before Filtration	After Filtration	Before Filtration	After Filtration
PUR + 5% CuO nm	96.8	86.9	62.7	30.9
PUR + 7% CuO nm	99.8	91.2	98.2	80
PUR + 9.5% CuO nm	100	96.8	98.8	78.3
PUR + 12% CuO nm	100	88.7	99.6	79.1

## Data Availability

The data presented in this study are available in supplementary material.

## Supplementary Materials

The following are available online, Figure S1: Content of microparticles of CuO on nanofiber’s surface before and after treatment under the simulated conditions of water filtration according to the results of EDX analysis (experiments with the rotating needle electrode, Table S1: Differences of antibacterial activity of samples with microparticles of CuO before and after treatment under the simulated conditions of water filtration (experiments with electrode with needle surface).

## Sample Availability

Samples of the compounds are not available from the authors.
