# Supplementary material for: Modified Nanofibrous Filters with Durable Antibacterial Properties
Source: Molecules. 2021 Feb 26;26(5):1255. doi: 10.3390/molecules26051255 (PMC7956445; doi:10.3390/molecules26051255)
Supplement: Supplementary file 1 [file molecules-26-01255-s001.pdf]

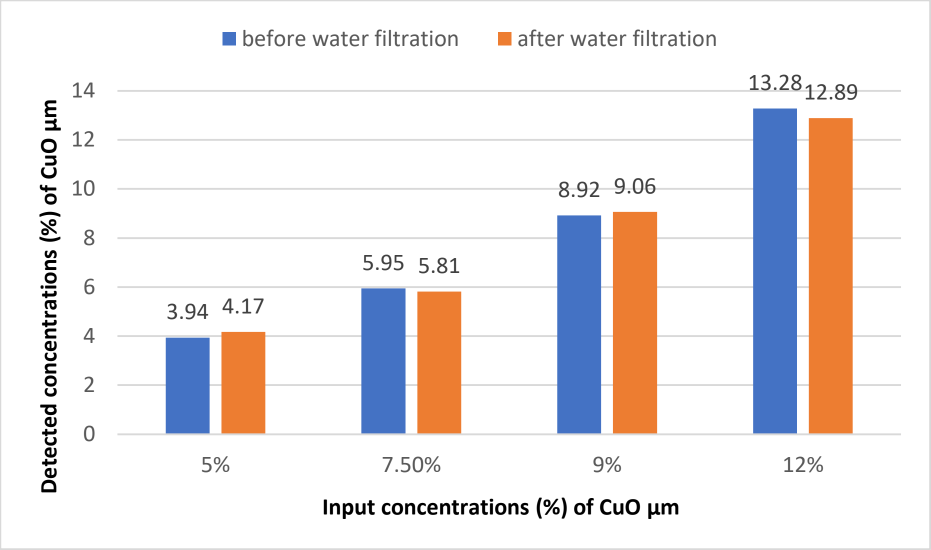

Figure S1. Content of microparticles of CuO on nanofiber's surface before and after treatment under the simulated conditions of water filtration according to the results of EDX analysis (experiments with the rotating needle electrode).

Table S1. Differences of antibacterial activity of samples with microparticles of CuO before and after treatment under the simulated conditions of water filtration (experiments with electrode with needle surface).

**Tested sample**	**Efficiency (%) - Escherichia coli**		**Efficiency (%) - Staphylococcus gallinarum**	
	**before filtration**	**after filtration**	**before filtration**	**after filtration**
PUR + 5% CuO µm	**97**	**97.3**	**98.8**	**97.9**
PUR + 7% CuO µm	**99.7**	**99.1**	**100**	**99.8**
PUR + 9.5% CuO µm	**100**	**100**	**100**	**100**
PUR + 12% CuO µm	**100**	**100**	**100**	**100**
